# HGF Secreted by Mesenchymal Stromal Cells Promotes Primordial Follicle Activation by Increasing the Activity of the PI3K-AKT Signaling Pathway

**DOI:** 10.1007/s12015-022-10335-x

**Published:** 2022-01-28

**Authors:** Xin Mi, Wenlin Jiao, Yajuan Yang, Yingying Qin, Zi-Jiang Chen, Shidou Zhao

**Affiliations:** 1 Center for Reproductive Medicine, Cheeloo College of Medicine, Shandong University, 44 Wenhua Xi Road, Jinan, 250012 Shandong China; 2 Key Laboratory of Reproductive Endocrinology of Ministry of Education, Shandong University, Jinan, 250012 Shandong China; 3 Shandong Key Laboratory of Reproductive Medicine, Jinan, 250012 Shandong China; 4 Shandong Provincial Clinical Research Center for Reproductive Health, Jinan, 250012 Shandong China; 5 National Research Center for Assisted Reproductive Technology and Reproductive Genetics, Shandong University, Jinan, 250012 Shandong China; 6 Shanghai Key Laboratory for Assisted Reproduction and Reproductive Genetics, Shanghai, 200135 China; 7 Center for Reproductive Medicine, Ren Ji Hospital, School of Medicine, Shanghai Jiao Tong University, Shanghai, 200135 China

**Keywords:** Mesenchymal stromal cells, Hepatocyte growth factor, Primordial follicle activation, Premature ovarian insufficiency

## Abstract

Primordial follicle activation is fundamental for folliculogenesis and for the maintenance of fertility. An effective therapeutic strategy for patients with premature ovarian insufficiency (POI) is to promote the activation of residual primordial follicles. The secretome of human umbilical cord mesenchymal stromal cells (hUC-MSC-sec) contains several components that might promote the activation of primordial follicles. In the present study, we revealed that treatment with the hUC-MSC-sec significantly increased the proportion of activated primordial follicles in mouse ovaries both in vitro and in vivo. The activating effects of hUC-MSC-sec on primordial follicles were attributed to the activation of the PI3K-AKT signaling pathway by hepatocyte growth factor (HGF). While the effect of the hUC-MSC-sec was attenuated by the neutralizing antibodies against HGF, application of exogenous HGF alone also promoted the activation of primordial follicles. Furthermore, we demonstrated that HGF promoted the expression of KITL in granulosa cells by binding with the HGF receptor c-Met, thereby increasing the activity of the PI3K-AKT signaling pathway to activate primordial follicles. Taken together, our findings demonstrate that hUC-MSC-sec promotes primordial follicle activation through the functional component HGF to increase the PI3K-AKT signaling activity, highlighting the application of the hUC-MSC-sec or HGF for the treatment of POI patients.

**Graphical abstract:**

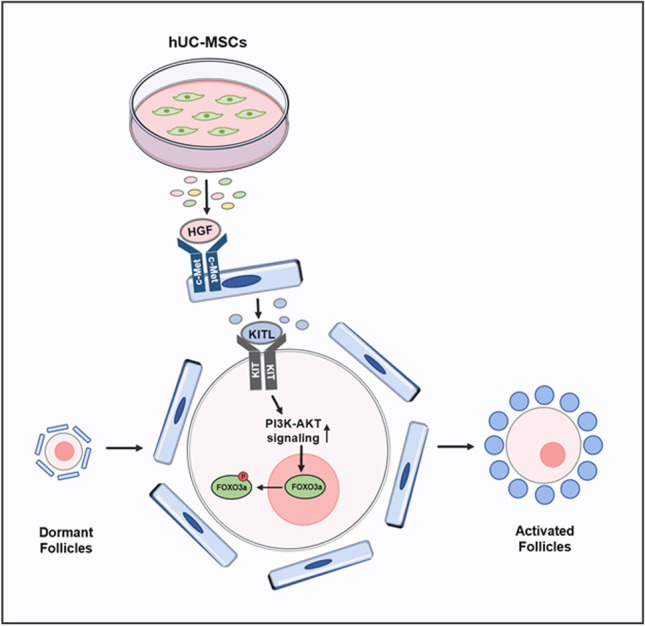

**Supplementary Information:**

The online version contains supplementary material available at 10.1007/s12015-022-10335-x.

## Background

The majority of follicles in the mammalian ovary are quiescent, forming the primordial follicle pool, and only a few are gradually recruited into the growth phase. [1]. The process that awakens the quiescent follicles is called primordial follicle activation, and this process continues throughout a woman's life and is fundamental for folliculogenesis and fertility [2, 3]. The predominant molecular mechanism for primordial follicle activation is the PI3K-AKT-FOXO3a signaling pathway, and this has been confirmed in multiple genetically modified mouse models [4, 5]. In recent years, the role of communication between granulosa cells and oocytes during primordial follicle activation has also been highlighted, especially the KIT-PI3K signaling in which the receptor tyrosine kinase KIT on the oocyte surface acts as an intermediary to activate the downstream PI3K signaling pathway after binding with KIT ligand (KITL) released by granulosa cells [6].

Premature ovarian insufficiency (POI) is defined as the decline of ovarian activity before the age of 40 years and is characterized by hormone imbalances, menstrual disorders, and infertility [7, 8]. For a long time, the only available treatment for infertility of POI patients has been oocyte donation [7]. However, recent studies have found that even POI patients with amenorrhea still have some residual primordial follicles in their ovaries, and this provides a new direction for the treatment of POI patients by promoting the activation of these primordial follicles [9, 10]. Based on this hypothesis, Kawamura et al. pioneered in vitro activation (IVA) of primordial follicles by treating human ovarian cortical fragments with PI3K-AKT pathway stimulators in vitro to promote the activation of dormant follicles [11]. This approach has now been applied clinically and has resulted in several live births [12]. However, considering the potential carcinogenicity of PI3K-AKT pathway stimulators and the invasive surgery, the prospects for the IVA technology are limited, and more effective and safe treatments for patients with POI are needed [13].

Due to their unique advantages, mesenchymal stromal cells (MSCs) have promising applications in many refractory diseases and are considered to be a new approach to treat ovarian damage and ovarian aging [14, 15]. It has been demonstrated that MSCs can improve the ovarian niche through paracrine effects and thus rescue ovarian function by promoting granulosa cell proliferation, ovarian angiogenesis, and oocyte maturation [16]. Human umbilical cord mesenchymal stromal cells (hUC-MSCs) are multipotent stem cells with high proliferation ability, high secretion capacity, and low immunogenicity, and these cells are free from the ethical issues [17–19]. Notably, using an ovary-collagen/hUC-MSC co-culture system, Ding et al. found that hUC-MSCs could promote the phosphorylation of AKT and FOXO3a in mouse ovaries in vitro, suggesting that hUC-MSCs might be able to promote primordial follicle activation [20]. However, the specific functional components and molecular mechanisms involved in promoting primordial follicle activation by hUC-MSCs remain unclear.

In the present study, we found that the hUC-MSC secretome (hUC-MSC-sec) could effectively promote the activation of primordial follicles both in vitro and in vivo. We further demonstrated that hepatocyte growth factor (HGF) secreted from hUC-MSCs plays an essential role during this process. Mechanistically, HGF promoted the release of KITL by combining with the HGF receptor c-Met on granulosa cells, thereby increasing the activity of the PI3K-AKT signaling in dormant oocytes. Our study clarifies the molecular mechanism and functional component of the hUC-MSC-sec to activate primordial follicles, and will provide a new approach for the treatment of POI.

## Materials and Methods

### Animals

Adult male and female C57BL/6 J mice (6 to 8 weeks old) were obtained from the Laboratory Animal Center of Shandong University. Female and male mice were mated overnight in a 2:1 ratio. Postnatal day 1 (PD1) was defined as the day after partum. Ovaries of PD5 females were used for ovarian in vitro culture. Adult female C57BL/6 J mice (PD35) used for ovarian in situ injection were purchased from Beijing Vital River Laboratory Animal Technology Co. Ltd.

### Isolation, Culture, and Characterization of hUC-MSCs

The hUC-MSCs were isolated by our laboratory and human tissue samples were handled in accordance with the National Regulation of Clinical Sampling in China. Briefly, human umbilical cord tissue was obtained and washed by PBS contained 1% penicillin–streptomycin (Hyclone, SV30010). The umbilical cord was then dissected into 3–4 cm pieces, and all the vessels were mechanically removed. Wharton's jelly from the umbilical cord was carefully peeled off and cut into 1–3 mm pieces and centrifuged at 400 × *g* for 5 min in PBS. The tissue segments were then cultured in Dulbecco’s modified Eagle’s medium (DMEM, GIBCO, 12571–063) supplemented with 5% GRO (Helios Ultra GRO, HPCFDCRL05) in a 37℃ humidified environment with 5% CO_2_. The first colony of hUC-MSCs was observed after approximately 7 days.

The cell surface antigens of hUC-MSCs were analyzed by flow cytometry using phycoerythrin (PE)-conjugated human monoclonal antibodies against CD90 (eBioscience, 11–0909-41), CD105 (eBioscience, 12–1057-41), CD73 (eBioscience, 11–0739-41), CD45 (eBioscience, 11–0459-41), and HLA-DR (eBioscience, 11–9952-41). Mouse IgG isotype (eBioscience, 12–4714-81) was used as the negative control.

### hUC-MSC-sec Preparation

hUC-MSCs between passages 6 and 9 were cultured in 75 cm^2^ cell culture flasks (Corning, 430641). After cells reached 90% confluency, the medium was replaced by DMEM/F12 medium (GIBCO, 11320–033) for an additional 48 h. Afterwards, the collected supernatant was centrifuged at 1000 × *g* for 5 min and filtered through a 0.22 μm filter. Afterward, ultra-filtration centrifuge tubes (3 KDa, Millipore, UFC900308) were used to concentrate the medium 25-fold by centrifugation at 5000 × *g* for 40–50 min. The concentrated hUC-MSC-sec was collected and stored at –80 °C or directly used for the subsequent experiments.

### Ovary Culture

Female mice were sacrificed by cervical dislocation at PD5. Mouse ovaries were separated under aseptic conditions in pre-warmed Leibovitz's-15 medium (GIBCO, 11415064) containing 10% fetal bovine serum (Biological Industries, C04001) and 1% penicillin–streptomycin. Isolated ovaries were randomly distributed with each group containing four to six ovaries and cultured on cell culture inserts (Millipore, MPICM0RG50) in 6-well culture plates with 1.2 ml DMEM/F12 medium (GIBCO, 11,320–033) plus 5% ITS (Sigma, I3146), 0.1 mol/l L-ascorbic acid (Sigma, A4403), 1 mg/ml bovine serum albumin (Sigma, B2064), 1 mg/ml Albumax II (Gibco, 11021029), and 1% penicillin–streptomycin. According to the experimental grouping, hUC-MSC-sec (tenfold concentrated), recombinant human HGF (800 ng/ml, Peprotech, 100–39), HGF antibody (1 μg/ml, R&D systems, AF-294), c-Met antibody (1 μg/ml, R&D systems, AF-276), or KITL antibody (1 μg/ml, R&D systems, AF-455) were added separately to the normal medium.

### Histological Staining and Follicle Counting

After fixing overnight in Bouin's solution (Sigma, HT10132), embedding in paraffin, and sectioning serially at 5 μm, ovarian sections were stained with hematoxylin and eosin. Follicles with a visible nucleus were counted in each of five sections of the whole ovary, and thus the final number was multiplied by a correction factor of 5. Total follicles included primordial follicles and activated follicles. Primordial follicles are identified as follicles with a small oocyte and one layer of flattened granulosa cells. Activated follicles are identified as follicles with one enlarged oocyte and a mixture of squamous and cuboidal granulosa cells surrounding it, or with one or several layers of cuboidal granulosa cells [2, 21]. The proportion of activated follicles was calculated as the number of activated follicles / the number of total follicles × 100%.

### Western Blot

Total proteins were extracted from cultured mouse ovaries (four included in each group) using the Total Protein Extraction Kit (Invent, SD-001/SN-002). After heating at 100 °C with SDS loading buffer for 10 min, total proteins were separated by SDS-PAGE and electrotransferred to the PVDF membrane. Membranes were blocked in TBST with 5% non-fat milk and then incubated with primary antibodies against p-AKT (CST, 9271), AKT (CST, 9272), KITL (Santa Cruz Biotechnology, sc-13126), and β-actin (Proteintech, 66009–1) at 4 °C overnight. β-Actin was used as the internal control. The membranes were subsequently incubated with HRP-conjugated secondary antibodies, and the protein bands were detected with a ChemiDoc MP System (Bio‐Rad).

### Immunofluorescence

In vitro cultured mouse ovaries (*n* = 6) were fixed in 4% PFA overnight, embedded in Frozen Section Medium (Thermo Scientific, Neg-50), frozen in liquid nitrogen, and serially sectioned at 10 μm. The sections were permeabilized and blocked by incubating with 0.3% Triton X‐100 and 25% donkey serum for 1 h. Then primary antibodies against FOXO3a (Rabbit IgG, CST, 2497) and DDX4 (Goat IgG, R&D system, AF2030) were incubated with the sections at 4 °C overnight. Afterwards, donkey anti-goat secondary antibody conjugated with Alexa Fluor 488, donkey anti-rabbit secondary antibody conjugated with Alexa Fluor 569 and 5 μg/ml Hoechst 33342 were incubated for 1 h at room temperature. Images were observed and captured under a fluorescence microscope (Olympus BX53). Cellular localization of FOXO3a was determined by costaining with the oocyte cytoplasmic marker DDX4 and the nuclear dye Hochest 33342. The proportion of oocytes with cytoplasmic localization of FOXO3a (CL-FOXO3a) was calculated as the number of oocytes with CL-FOXO3a / number of FOXO3a-positive oocytes × 100%.

### Immunohistochemistry

Ovaries from PD5 mice were fixed in Bouin’s solution overnight, embedded in paraffin, and sectioned serially at 5 μm. After deparaffinization and rehydration, the sections were boiled in EDTA solution (pH 8.0) for 40 min to retrieve the antigen. Then the sections were permeabilized and blocked by incubating with 0.3% Triton X‐100 and 10% bovine serum albumin for 1 h. The sections were then incubated with c-Met antibody (c-Met^ab^, Santa Cruz Biotechnology, sc-8057) at 4 °C overnight. Normal mouse IgG (Santa Cruz Biotechnology, sc-2025) was used as a negative control. On the second day, all sections were incubated with goat anti-mouse secondary antibody and stained with DAB (Vectorlabs, SK-4100). The nuclei were counterstained with hematoxylin and then observed under the microscope.

### KGN Cell Culture

The human granulosa-like tumor cell line KGN (RIKEN BioResource Center, Japan) [22] was cultured in DMEM/F12 medium containing 10% fetal bovine serum and 1% penicillin–streptomycin at 37 °C in a humidified 5% CO_2_ incubator. According to the experimental grouping, recombinant human HGF (800 ng/ml, Peprotech, 100–39) with or without c-Met^ab^ (1 μg/ml, R&D systems, AF-276) were added to the normal medium for 48 h before protein extraction.

### Ovarian In Situ Injection

Twenty-four adult female mice (PD35) were randomly assigned equally into the following four groups: the control group (PBS injection), the hUC-MSC-sec group (30-fold concentrated hUC-MSC-sec injection), the hUC-MSC-sec + HGF^ab^ group (30-fold concentrated hUC-MSC-sec plus 1 μg/ml HGF^ab^ injection), and the HGF group (800 ng/ml HGF injection). Autocrosslinked hyaluronan gel (0.3 mg/ml, Bioregen) was added to each group as a drug carrier. The mice were anesthetized, and their ovaries were gently exposed from incisions through their backs. After injecting 8 μl of solution into each ovary in situ with a microsyringe, the incisions were sutured. The mice were then maintained under standard conditions for 14 days prior to sacrifice.

### Statistical Analysis

The statistical analyses were performed with SPSS 25.0 software, and all numerical values are presented as mean ± SD. Student’s t-test was used for comparisons between two groups, and one-way analysis of variance (ANOVA) with LSD multiple comparison analysis was used for comparisons of three or more groups. The difference was considered to be statistically significant when *P* < 0.05.

## Results

### The hUC-MSC-sec Promoted Primordial Follicle Activation In Vitro

The hUC-MSCs used in this study were characterized by flow cytometry. The results showed that the cells with the expression of CD90, CD73, and CD105 were 100%, 100%, and 99.8%, respectively. In contrast, the cells with the expression of negative markers CD45 and HLA-DR were both lower than 1% (Fig. S1).

**Fig. 1:**
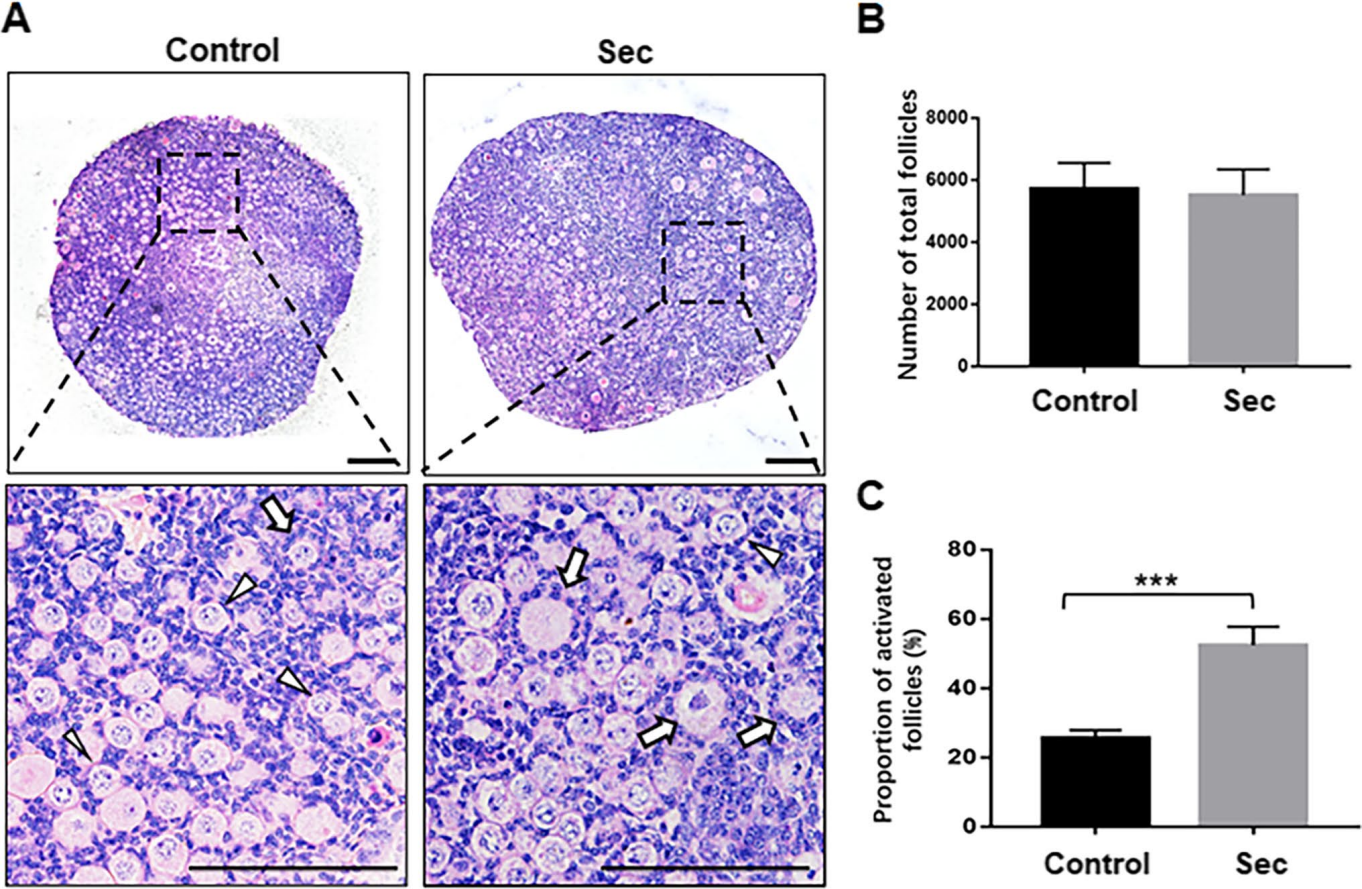
The hUC-MSC-sec promoted primordial follicle activation in vitro*.* **A.** After 12 days of in vitro culture, the histological analysis of hematoxylin and eosin staining showed more activated follicles in hUC-MSC-sec-treated ovaries than controls. The arrowheads indicate primordial follicles, and the arrows indicate activated follicles. **B.** Quantification of ovarian follicles showed no obvious difference in the total number of follicles between hUC-MSC-sec-treated ovaries (5510 ± 832.2) and controls (5723 ± 825.3). **C.** Ovarian follicle counts revealed a significantly increased proportion of activated follicles in hUC-MSC-sec-treated ovaries (52.4 ± 5.3%) compared to controls (25.5 ± 2.3%). Data are shown as the mean ± SD, *n* = 6. ***P < 0.001. Scale bars, 100 μm

It is known that mouse ovary at PD3 only contains primordial follicles which are activated thereafter, and few well-developed primary follicles appear in ovarian medulla at PD7 [1, 23]. Therefore, the period between PD3 and PD7 with the dominance of primordial follicles is considered as the appropriate time to study primordial follicle activation in mice. The whole ovary culture of neonatal mice, which preserves the normal interaction between oocytes and ovarian somatic cells, has been widely used for the study of primordial follicle activation [21, 24]. In this study, to evaluate the effect of the hUC-MSC-sec, ovaries from PD5 mice were cultured in vitro with or without the hUC-MSC-sec. After 12 days, a notable increase in ovarian size and more activated follicles were observed in hUC-MSC-sec-treated ovaries (Fig. 1A). While the total number of follicles in MSC-sec-treated ovaries was not obviously different from the control group (Fig. 1B), the proportion of activated follicles was significantly increased (Fig. 1C), suggesting that hUC-MSC-sec treatment promotes the activation of primordial follicles.

### The hUC-MSC-sec Increased the Activation of the PI3K-AKT Signaling Pathway

**Fig. 2:**
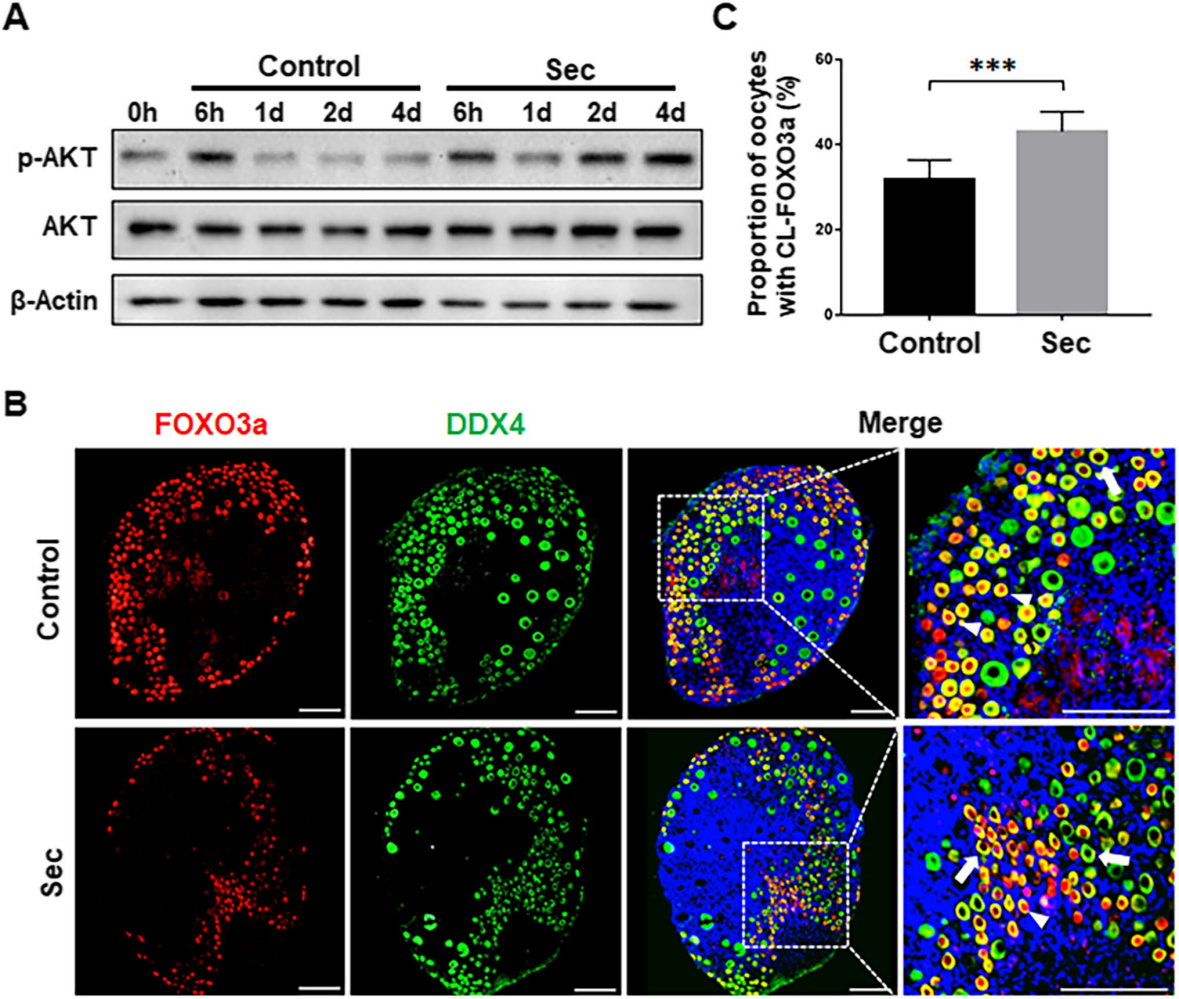
The hUC-MSC-sec activated the PI3K-AKT signaling pathway. **A.** There was a time-dependent increase in p-AKT level in hUC-MSC-sec-treated ovaries after 1, 2, and 4 days of in vitro culture compared to controls. β-Actin was used as the internal control. **B.** The localization of FOXO3a (red fluorescence) in oocyte cytoplasm (DDX4, green fluorescence) was increased in MSC-sec-treated ovaries after 4 days of culture compared to controls. Nuclei were counterstained with Hoechst 33342 (blue fluorescence). The arrowheads indicate nuclear localization of FOXO3a, and the arrows indicate the cytoplasmic localization of FOXO3a. **C.** The proportion of cytoplasmic localization of FOXO3a (CL-FOXO3a) was significantly increased in MSC-sec-treated ovaries (43.0 ± 4.7%) compared to controls (31.8 ± 4.6%). Data are shown as the mean ± SD, *n* = 6. ***P < 0.001. Scale bars, 100 μm

To determine whether the hUC-MSC-sec could promote the activation of the PI3K-AKT pathway, ovaries from PD5 mice were cultured for 6 h, 1 day, 2 days, or 4 days. Western blot analysis showed that at 6 h the phosphorylation of Akt was significantly increased in both the hUC-MSC-sec and control groups compared with 0 h, which might reflect the transient activation of the PI3K-AKT pathway due to the surgical procedure itself [25]. The phosphorylation of AKT was gradually increased in the hUC-MSC-sec-treated ovaries compared with the controls from the first day onwards without any significant changes in total AKT (Fig. 2A).

We next determined the localization of FOXO3a by immunofluorescence staining in ovaries that had been cultured for 4 days. FOXO3a is downstream of the PI3K-AKT pathway and localizes in the nucleus of oocytes to maintain primordial follicles in a quiescent state. After activation of the PI3K-AKT signaling pathway, FOXO3a is phosphorylated and then translocates from the nucleus into the cytoplasm, thus relieving its inhibitory effect and resulting in primordial follicle activation [26]. The results showed an increased proportion of oocytes with cytoplasmic localization of FOXO3a in hUC-MSC-sec-treated ovaries compared to the controls (Fig. 2B and C). These results demonstrated that hUC-MSC-sec treatment effectively promoted the activation of the PI3K-AKT-FOXO3a signaling pathway.

### HGF Secreted from hUC-MSCs Promoted the Activation of the PI3K-AKT Pathway

**Fig. 3:**
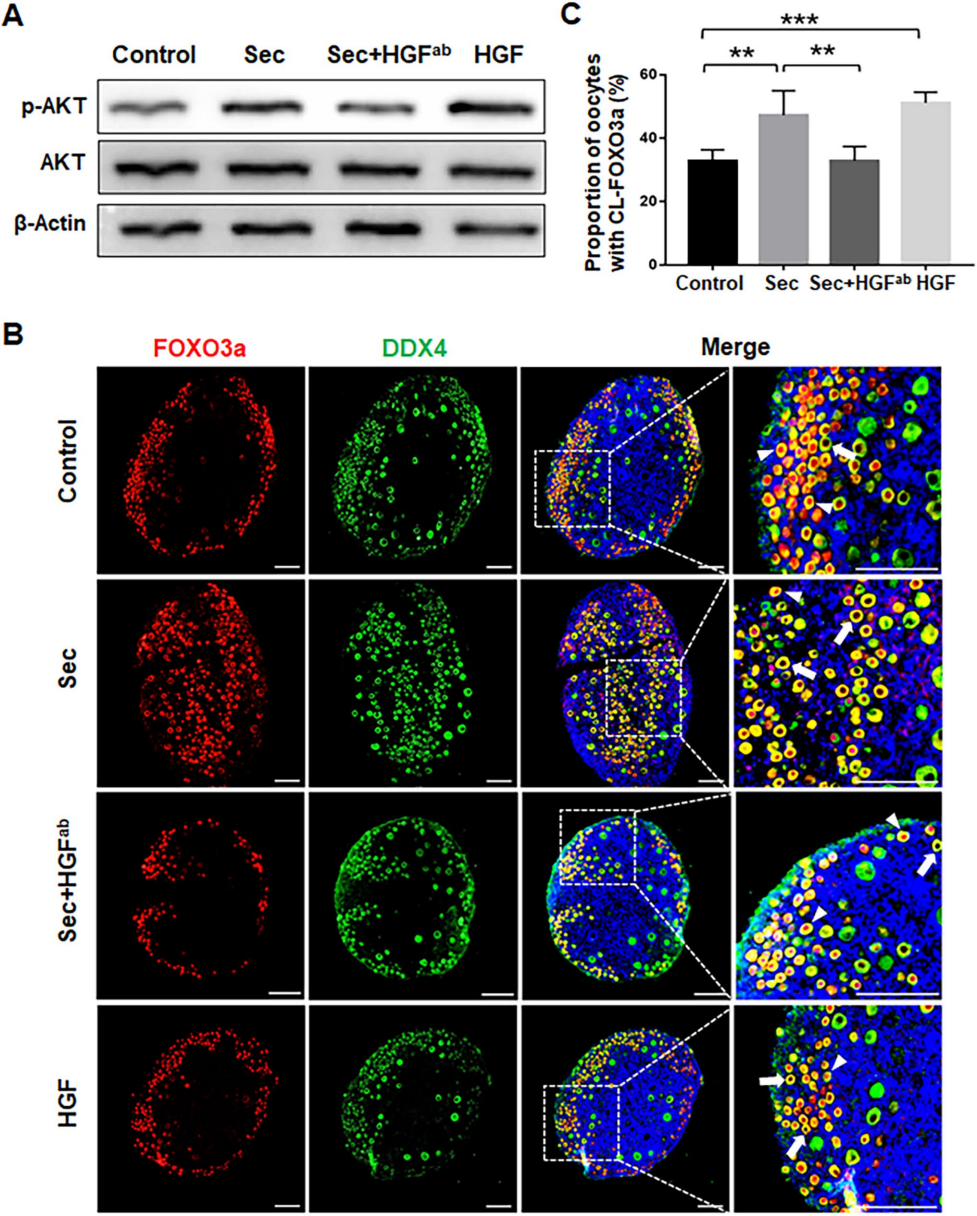
HGF secreted from hUC-MSCs promoted the activation of the PI3K-AKT pathway. **A.** Western blot showing the expression of p-AKT in mouse ovaries after 4 days of in vitro culture. The ability of the hUC-MSC-sec to increase the phosphorylation of AKT was greatly inhibited by the addition of HGF^ab^. The expression of p-AKT was significantly increased in the HGF-treated group compared to controls. **B.** Immunofluorescence analysis showing the location of FOXO3a in mouse ovaries after 4 days of culture. The ability of the hUC-MSC-sec to promote FOXO3a cytoplasmic translocation was inhibited by the addition of HGF^ab^. The arrowheads indicate the nuclear localization of FOXO3a, and the arrows indicate the cytoplasmic localization of FOXO3a. **C.** The proportion of CL-FOXO3a was significantly decreased in hUC-MSC-sec plus HGF^ab^-treated ovaries (32.8 ± 4.6%) compared to the hUC-MSC-sec group (47.3 ± 7.7%). The proportion of CL-FOXO3a was significantly increased in HGF-treated ovaries (50.9 ± 3.1%), which was similar to the hUC-MSC-sec group, compared to controls (32.8 ± 3.5%). Data are shown as the mean ± SD, n = 6. ^**^P < 0.01, and ^***^P < 0.001. Scale bars, 100 μm

After confirming the effects of the hUC-MSC-sec on primordial follicle activation, we focused on exploring the functional component that mediates this effect. The hUC-MSC-sec contains a variety of soluble factors and extracellular vesicles [27], and proteomic analysis has shown that the soluble factors secreted by MSCs contain angiogenic factors, growth factors, chemokines, cytokines, etc. [28, 29]. By using a cytokine array, our previous study showed that HGF was the top one with much more content in MSC-sec than that in the secretome of fibroblasts and was identified as the effective component to promote primordial follicle survival by activating the PI3K-AKT pathway [30]. Similarly, Jia et al. also found a high content of HGF in hUC-MSC-sec by using human cytokine antibody array [31]. It is speculated that HGF might be a functional component of the hUC-MSC-sec that promotes the activation of primordial follicles [32]. To determine the role of HGF in the hUC-MSC-sec, a neutralizing antibody against HGF (HGF^ab^) was added to the hUC-MSC-sec, and this reduced the levels of phosphorylated Akt as well as the proportion of oocytes with cytoplasmic localization of FOXO3a compared to the hUC-MSC-sec group (Fig. 3A-C). Consistent with these findings, we also used exogenous HGF alone and found similar results as for hUC-MSC-sec, i.e. the application of HGF also promoted the activation of the PI3K-AKT-FOXO3a signaling pathway (Fig. 3A-C). Taken together, these results revealed that HGF is one of the functional components of the hUC-MSC-sec that promotes primordial follicle activation.

### The hUC-MSC-sec Promoted the Activation of the PI3K-AKT Pathway Through c-Met on Granulosa Cells

**Fig. 4:**
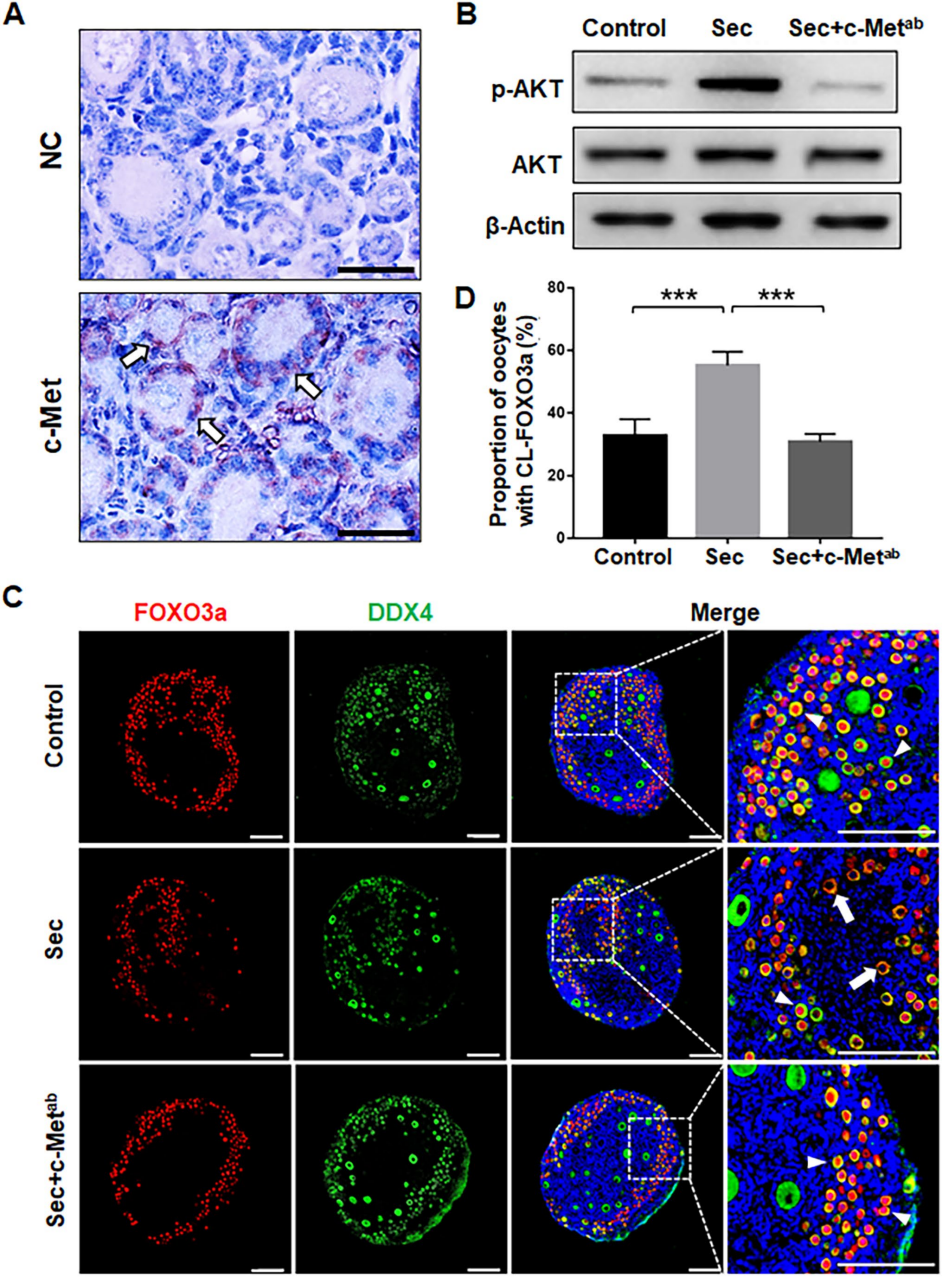
The hUC-MSC-sec promoted the activation of the PI3K-AKT pathway through c-Met on the granulosa cells. **A.** Immunostaining showing that c-Met is expressed on granulosa cells in both primordial and activated follicles. Normal mouse IgG was used in place of the primary antibody for the negative control. **B.** Western blot showing the expression of p-AKT in mouse ovaries after 4 days of in vitro culture. The ability of the MSC-sec to increase the phosphorylation of AKT was greatly inhibited by the addition of c-Met^ab^. **C.** Immunofluorescence analysis showing the location of FOXO3a in mouse ovaries after 4 days of in vitro culture. The ability of the hUC-MSC-sec to promote translocation of FOXO3a from the nucleus to the cytoplasm was inhibited by the addition of c-Met^ab^. The arrowheads indicate the nuclear localization of FOXO3a, and the arrows indicate the cytoplasmic localization of FOXO3a. **D.** The proportion of CL-FOXO3a was significantly decreased in hUC-MSC-sec plus c-Met^ab^-treated ovaries (30.8 ± 2.5%), which was similar to controls (32.7 ± 5.3%), compared to the hUC-MSC-sec group (55.2 ± 4.4%). Data are shown as the mean ± SD, *n* = 6. ***P < 0.001. Scale bars in A, 20 μm; Scale bars in C, 100 μm

To further investigate the mechanism through which the hUC-MSC-sec activates primordial follicles, we examined the expression and localization of the HGF receptor, c-Met, in the ovary. Immunohistochemistry analysis showed that it was predominantly expressed on granulosa cells both in primordial and activated follicles in PD5 ovaries (Fig. 4A).

To further determine whether the hUC-MSC-sec acts through binding to the c-Met receptor, c-Met^ab^ was added to the hUC-MSC-sec to block receptor-ligand interactions. The results showed that both the phosphorylation of Akt and the proportion of oocytes with cytoplasmic localization of FOXO3a were greatly decreased compared to the hUC-MSC-sec group (Fig. 4B-D). In addition, the effect of HGF to increase the proportion of activated oocytes with cytoplasmic localization of FOXO3a was blocked by c-Met^ab^ (Fig. S2). These results suggest that c-Met localized on granulosa cells is the specific receptor for HGF to promote the activation of the PI3K-AKT pathway.

### HGF Promoted the Activation of the PI3K-AKT Pathway by Increasing KITL Expression

**Fig. 5:**
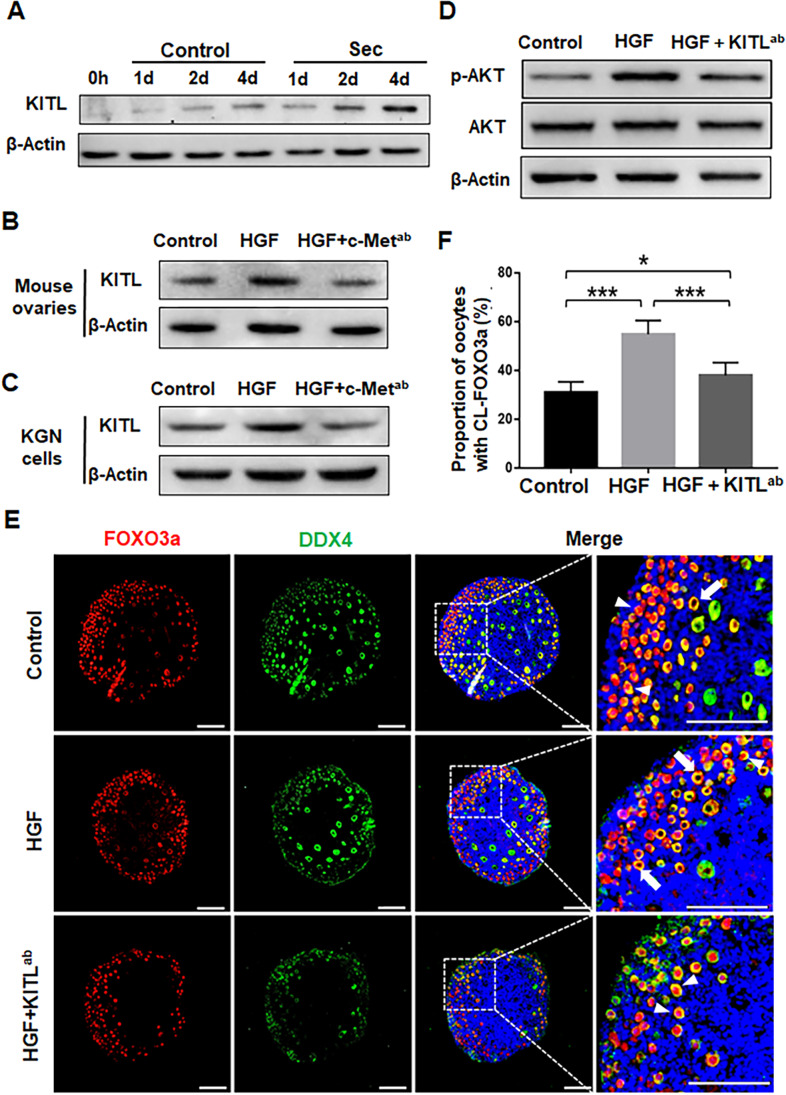
HGF promoted the activation of the PI3K-AKT pathway by increasing KITL expression. **A.** There was a time-dependent increase in KITL expression in hUC-MSC-sec-treated ovaries after 1, 2, and 4 days of in vitro culture compared to controls. β-actin was used as the internal control. **B.** Western blot showed that the expression of KITL was increased in HGF-treated mouse ovaries compared to controls, and the effect of HGF was blocked by the addition of c-Met^ab^. **C.** Western blot showed that the expression of KITL was increased in HGF-treated KGN cells compared to controls, and the effect of HGF was blocked by the addition of c-Met^ab^. **D.** Western blot showing the expression of p-AKT in mouse ovaries after 4 days of in vitro culture. The ability of HGF to increase the phosphorylation of AKT was greatly inhibited by the addition of KITL^ab^. **E.** Immunofluorescence analysis showing the location of FOXO3a in mouse ovaries after 4 days in vitro culture. The ability of HGF to promote FOXO3a cytoplasmic translocation was inhibited by the addition of KITL^ab^. The arrowhead indicates the nuclear localization of FOXO3a, and the arrow indicates the cytoplasmic localization of FOXO3a. **F.** The proportion of CL-FOXO3a was significantly decreased in ovaries with HGF and KITL^ab^ (37.9 ± 5.2%) compared to the HGF group (54.8 ± 5.7%), but it was still increased compared with controls (31.1 ± 4.2%). Data are shown as the mean ± SD, *n* = 6. *P < 0.05, and ***P < 0.001. Scale bars, 100 μm

It has been reported that HGF promotes KITL secretion in mouse granulosa cells [33, 34]. To further explore the molecular changes that occur in the granulosa cells after HGF binding to the c-Met receptor, we first measured the expression of KITL in ovaries cultured with hUC-MSC-sec for 1 day, 2 days, or 4 days. Western blot analysis showed a significant increase in KITL expression after 2 days and 4 days (Fig. 5A). Next, the cultured ovaries were incubated with HGF for 4 days, and the expression of KITL was increased. However, after adding c-Met^ab^ along with HGF, the promotion of KITL expression by HGF was blocked (Fig. 5B). We also obtained similar results in cultured KGN cells, which are a human granulosa-like tumor cell line (Fig. 5C), further confirming that HGF increases KITL expression in granulosa cells by binding to c-Met.

Next, we added KITL^ab^ in the presence of HGF to verify that the activation of the PI3K-AKT-FOXO3a pathway was due to the increase in KITL expression. Both phosphorylated Akt levels and the proportion of oocytes with FOXO3a cytoplasmic localization were significantly inhibited by the addition of KITL^ab^ compared to the HGF group (Fig. 5D-F). These results suggest that HGF, the functional component secreted by hUC-MSCs, promotes the expression of KITL to increase the activity of the PI3K-AKT signaling.

### HGF Promoted the Activation of Primordial Follicles via KITL

**Fig. 6:**
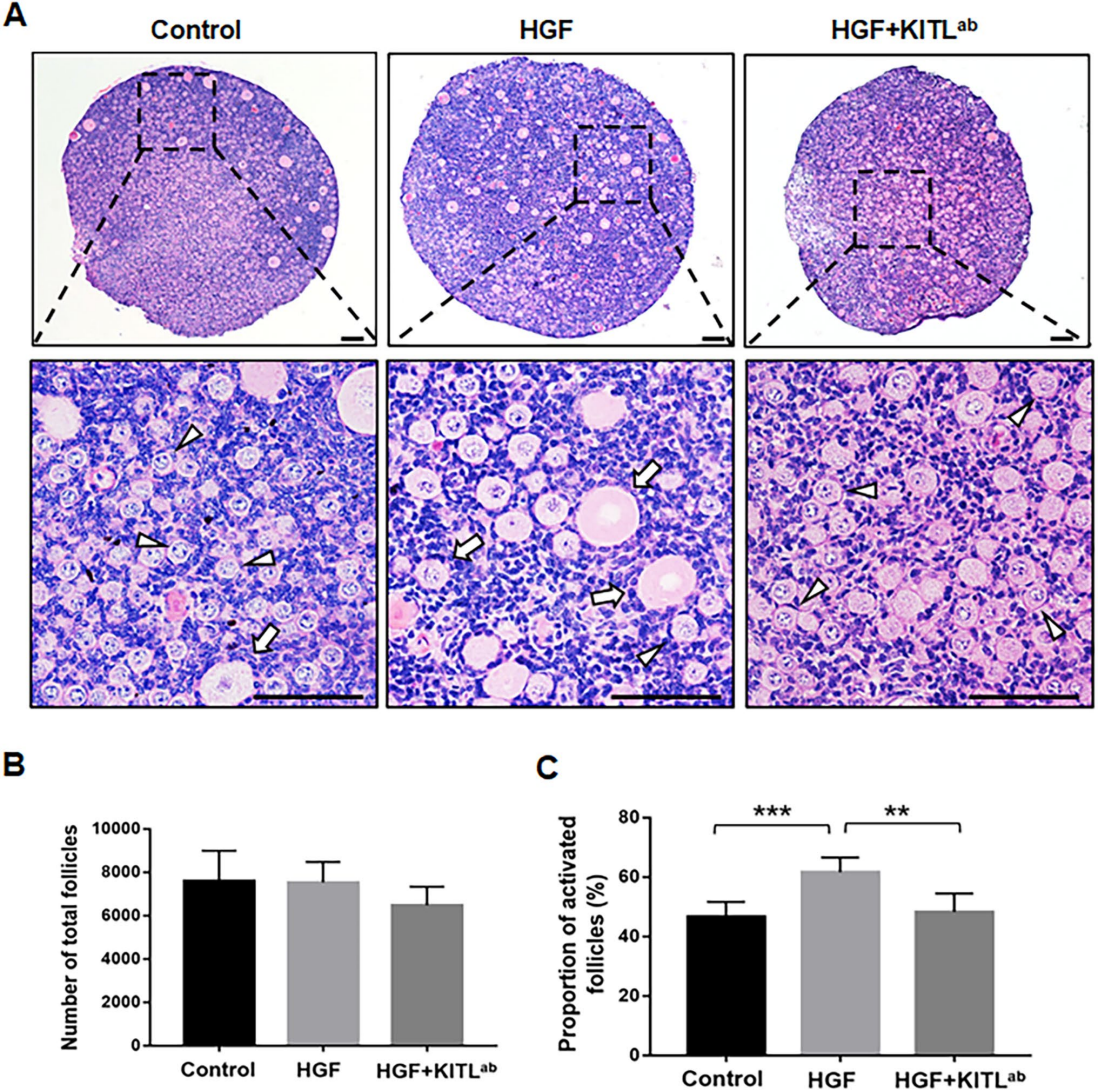
HGF promotes the activation of primordial follicles via KITL. **A.** After 12 days of in vitro culture, the histological staining showed more activated follicles in HGF-treated ovaries than controls. The number of activated follicles was decreased in the HGF plus KITL^ab^ group compared to the HGF group. The arrowheads indicate primordial follicles, and the arrows indicate activated follicles. **B.** Quantification of ovarian follicles showed no obvious differences in the total follicle count among the control group (7588 ± 1408), the HGF-treated group (7533 ± 945), and the HGF plus KITL^ab^-treated group (6472 ± 866.4). **C.** Quantification of ovarian follicles showed a significantly increased proportion of activated follicles in HGF-treated ovaries (61.6 ± 5.0%) compared to controls (46.0 ± 4.5%) and a significantly decreased proportion in HGF plus KITL^ab^-treated ovaries (48.1 ± 6.2%) compared to HGF-treated ovaries. Data are shown as the mean ± SD, *n* = 6. **P < 0.01, and ***P < 0.001. Scale bars, 50 μm

To further verify the activation of primordial follicles, HGF was added to cultured ovaries with or without KITL^ab^. After 12 days, while there was a large proportion of activated follicles in the HGF-treated group compared to the control group (Fig. 6A-C), the proportion of activated follicles was markedly decreased in ovaries treated with HGF and KITL^ab^ compared to ovaries treated with HGF alone (Fig. 6C). These results confirmed that, similar to the hUC-MSC-sec, HGF promotes primordial follicle activation in vitro via KITL.

### Ovarian Injection with the hUC-MSC-sec Promoted Primordial Follicle Activation In Vivo

**Fig. 7:**
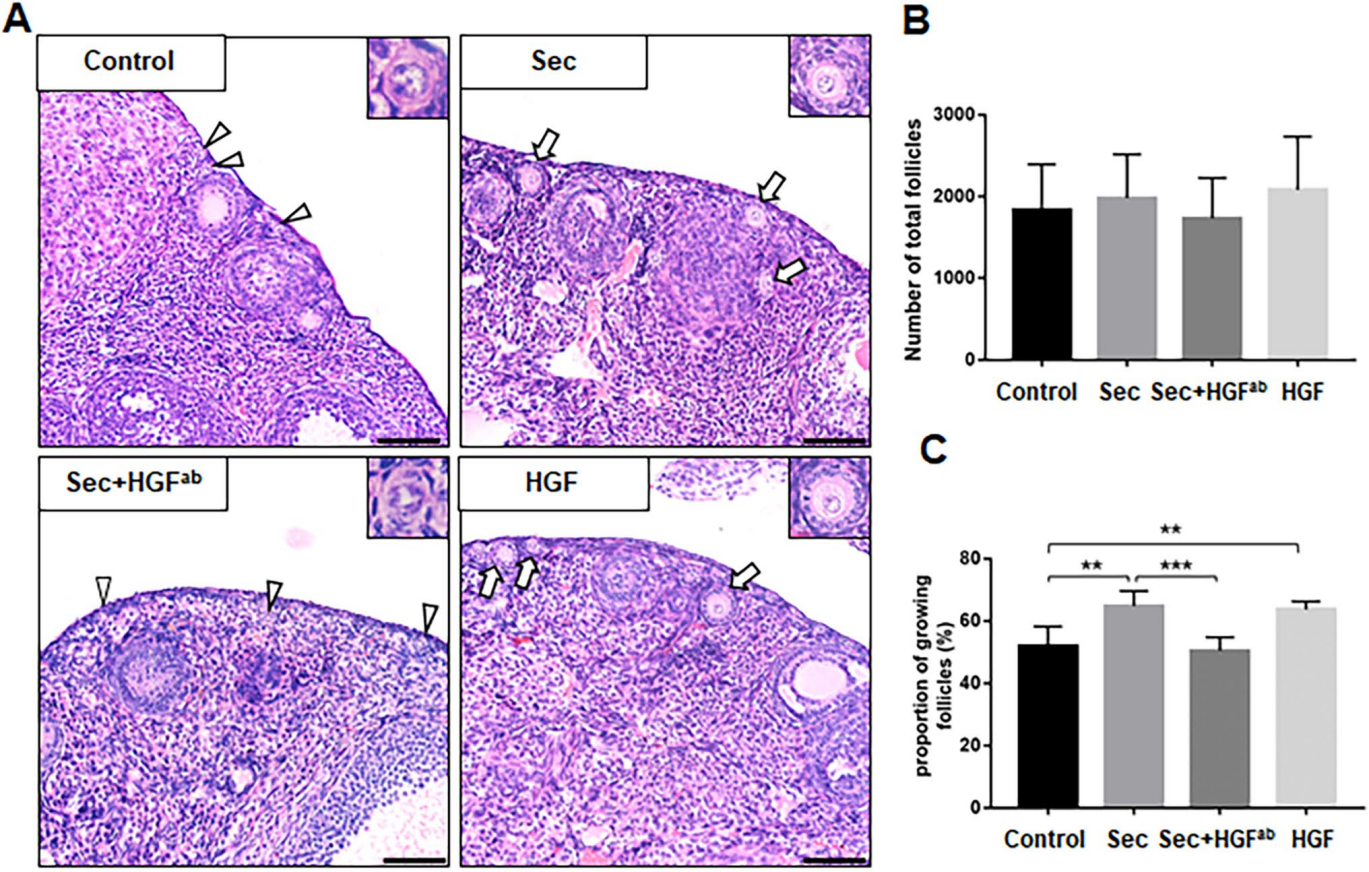
Ovarian injection of the hUC-MSC-sec promotes primordial follicle activation in vivo. **A.** After 14 days of in situ injection into adult female mice ovaries, the histological staining showed more growing follicles in the hUC-MSC-sec and HGF groups than in the control group, and fewer growing follicles in the hUC-MSC-sec plus HGF^ab^ group than in the hUC-MSC-sec group. The arrowheads indicate primordial follicles, and the arrows indicate growing follicles. **B.** Quantification of ovarian follicles showed no obvious differences in the total number of follicles among the control group (1833 ± 228.6), the hUC-MSC-sec group (1980 ± 239.6), the hUC-MSC-sec plus HGF^ab^ group (1730 ± 202.2), and the HGF group (2082 ± 266.7). **c.** Quantification of ovarian follicles showed a significantly increased proportion of activated follicles in the hUC-MSC-sec (64.8 ± 4.8%) and HGF groups (63.7 ± 2.6%) compared to the control group (51.9 ± 6.3%), and a significantly decreased proportion in the hUC-MSC-sec plus HGF^ab^ group (50.5 ± 4.3%) compared to the hUC-MSC-sec group. Data are shown as the mean ± SD, *n* = 6. **P < 0.01, and ***P < 0.001. Scale bars, 50 μm

Finally, in order to confirm the effects of hUC-MSC-sec and HGF on primordial follicle activation in vivo, we injected hUC-MSC-sec or HGF into adult mouse ovaries. To prolong the residence time after injection, we used autocrosslinked hyaluronan gel as the drug carrier [35]. The hUC-MSC-sec, HGF, or PBS was mixed separately with the autocrosslinked hyaluronan and injected into the ovaries of adult female mice. Histological observations showed an increased proportion of growing follicles after 2 weeks of hUC-MSC-sec or HGF injection compared to the control group (Fig. 7A-C), indicating that both the hUC-MSC-sec and HGF can promote primordial follicle activation in vivo. In addition, neutralization of HGF in the hUC-MSC-sec with HGF^ab^ led to significantly lower proportion of growing follicles (Fig. 7C), thus verifying that HGF is the functional component of the hUC-MSC-sec.

## Discussion

Primordial follicles follow three main fates, including activation and entrance into the growing follicle pool, maintenance of the quiescent state, or dying directly from the quiescent state [36]. The proper balance between primordial follicle quiescence and activation is essential for the maintenance of fertility. While overactivation of primordial follicles leads to premature depletion of the ovarian reserve, inactivation of primordial follicles adversely affects the establishment of the growing follicle pool [37]. Therefore, artificial methods of primordial follicle activation would be valuable for fertility interventions. Notably, the clinical application of IVA approach has shown that the activation of the PI3K-AKT signaling pathway is effective to promote follicle growth [11, 12]. The PI3K-AKT signaling pathway has received the most attention among the molecular mechanisms that govern primordial follicle activation [6]. Under physiological conditions, FOXO3a, which is a substrate of Akt, inhibits primordial follicle activation and maintains follicular quiescence. Deletion of the *FOXO3a* gene causes global follicular activation, early follicle depletion, and infertility [26, 38]. After FOXO3a phosphorylation, it translocates from nucleus to cytoplasm, which can be considered as an indicator of primordial follicle activation [26].

In the present study, we found that the hUC-MSC-sec can effectively promote primordial follicle activation via the PI3K-AKT-FOXO3a pathway. Over the last decade, the application of MSC-based therapy in ovarian aging and injury has been widely studied and has undergone vigorous development [16, 18]. Although MSC transplantation is considered to be safe, the heterogeneity between cells of different origins, the low survival rate of cells post-transplantation, and the risk of embolization should not be underestimated [39, 40]. Compared with the direct transplantation of live cells, the application of the MSC-sec has superior safety and manageability [41]. Evidence is mounting that MSCs mostly exert their therapeutic effects through paracrine functions, and secretome-based cell-free therapy has also shown a great potential [15, 16]. The MSC-sec has been demonstrated in numerous animal experiments to improve the cellular characteristics and microenvironment of the ovary by preventing apoptosis, promoting cell proliferation, inducing angiogenesis, mediating immunomodulation, and preventing oxidation and fibrosis [16]. Because the advantageous effects of the MSC-sec on damaged ovaries are multifaceted, we focused on determining the role and mechanism of the hUC-MSC-sec in primordial follicle activation under physiological conditions. Using the hUC-MSC-sec as an in vivo activator of primordial follicles would provide multiple benefits for POI patients.

By adding HGF^ab^ to the hUC-MSC-sec, we found that both activation of the PI3K-AKT pathway and the proportion of activated follicles were dramatically reduced, thus demonstrating the essential role for HGF secreted by hUC-MSCs in mediating the activation of primordial follicles. HGF, which is secreted by stromal cells from a variety of tissues, is a multifunctional growth factor that mediates various biological actions, such as cell proliferation, angiogenesis, and anti-apoptosis [32]. Within the ovary, HGF is mainly produced by theca cells and regulates the development of ovarian follicles, including the proliferation and growth of granulosa cells and steroidogenesis [42]. HGF is more abundant in follicular fluid (24.2 ± 1.2 ng/ml) than in serum (0.28 ± 0.04 ng/ml) [43]. According to the HGF/c-Met signaling system in ovarian follicles, the action of HGF is dependent on binding to the receptor tyrosine kinase c-Met, which is mainly localized on granulosa cells in the ovary [44, 45]. In this study, we also confirmed that c-Met is expressed on granulosa cells of both primordial and activated follicles. The c-Met^ab^ was added to the hUC-MSC-sec or together with HGF to block interactions between HGF and c-Met, thus demonstrating that c-Met is the specific receptor for HGF to activate the PI3K-AKT pathway. These results support the important role of HGF secreted by hUC-MSCs in mediating primordial follicle activation. In addition, the present study showed that the PI3K-AKT pathway and primordial follicle activation can be effectively promoted by the application of HGF alone.

**Fig. 8:**
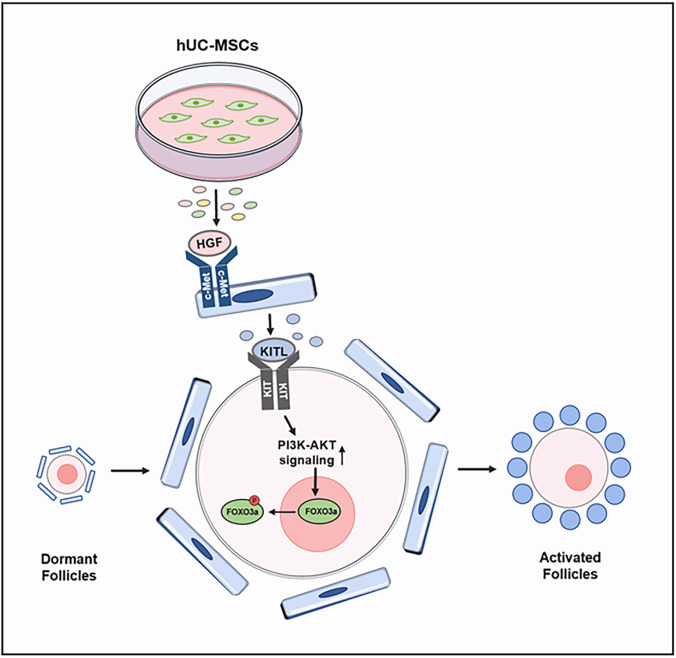
Molecular mechanisms through which the hUC-MSC-sec promotes primordial follicle activation. HGF secreted from MSCs promotes the expression of KITL, thereby enhancing the activity of the PI3K-AKT signaling pathway in dormant oocytes and thus activating primordial follicles

The interactive signaling between granulosa cells and oocytes is essential for folliculogenesis [46, 47]. In the early stage, KITL-KIT signaling plays a critical role in follicle growth as a communication between granulosa cells and oocytes [48]. After secretion by granulosa cells, KITL acts on the receptor tyrosine kinase KIT on the oocyte surface, to activate the PI3K-AKT pathway, which in turn mediates follicle activation [6]. In vitro experiments have demonstrated that the addition of HGF to isolated granulosa cells promotes the expression of KITL, and it is possible that endogenous HGF promotes KITL release to activate primordial follicles [33, 34]. In this study, we found that KITL expression was increased when adding the hUC-MSC-sec or HGF to cultured ovaries or granulosa cells. We also added KITL^ab^ to the HGF-treated group and found that both the activation of the PI3K-AKT pathway and the proportion of activated follicles were decreased, indicating that the increased expression of KITL is indispensable in this process. Based on the previous and present findings, we conclude that HGF secreted by MSCs promotes the expression of KITL by binding to c-Met on granulosa cells, thereby increasing the activity of the PI3K-AKT signaling pathway in dormant oocytes and promoting the activation of primordial follicles (Fig. 8).

While it has been demonstrated that the effective component HGF plays an essential role in MSC-sec-mediated primordial follicle activation, there may be other components that mediate the function of MSCs. Yang et al. cultured newborn ovaries with exosomes secreted from hUC-MSCs and demonstrated that miRNAs carried by exosomes, such as miR-146a-5p and miR-21-5p, mediated the activation of primordial follicles [49]. Moreover, we previously demonstrated that epidermal growth factor (EGF), which is also abundant in the MSC-sec [28, 50], could promote the activation of primordial follicles both in vivo and in vitro by increasing the CDC42-PI3K signaling activity [21, 51]. Therefore, the benefits of the MSC-sec for ovaries might be a combination of many components working together.

## Conclusions

In summary, we demonstrate that the hUC-MSC-sec can effectively promote primordial follicle activation both in vitro and in vivo, and we show that HGF secreted from hUC-MSCs promotes the expression of KITL in granulosa cells, thereby enhancing the activation of the PI3K-AKT signaling pathway in dormant oocytes. Our study clarifies the potential molecular mechanism for hUC-MSC-sec to activate primordial follicles, providing a theoretical basis for the clinical application of the hUC-MSC-sec or HGF for the treatment of POI patients.

## Supplementary Information

Below is the link to the electronic supplementary material.

Supplementary file1 (DOCX 1124 KB)

## Data Availability

The datasets used and/or analyzed during the current study are available from the corresponding author on reasonable request.

## Abbreviations

CL-FOXO3a: Cytoplasmic localization of FOXO3a
EGF: Epidermal growth factor
HGF: Hepatocyte growth factor
hUC-MSCs: Human umbilical cord mesenchymal stromal cells
hUC-MSC-sec: Human umbilical cord mesenchymal stromal cells’ secretome
IVA: In vitro activation
KITL: Kit ligand
POI: Premature ovarian insufficiency

## References

[1] McGee EA, Hsueh AJ (2000). Initial and cyclic recruitment of ovarian follicles. Endocrine Reviews.

[2] Picton HM (2001). Activation of follicle development: The primordial follicle. Theriogenology.

[3] Ford EA, Beckett EL, Roman SD, McLaughlin EA, Sutherland JM (2020). Advances in human primordial follicle activation and premature ovarian insufficiency. Reproduction.

[4] Cecconi S, Mauro A, Cellini V, Patacchiola F (2012). The role of Akt signalling in the mammalian ovary. International Journal of Developmental Biology.

[5] Reddy, P., Zheng, W., & Liu, K. (2010). Mechanisms maintaining the dormancy and survival of mammalian primordial follicles. *Trends in Endocrinology and Metabolism,**21*(2), 96–103. 10.1016/j.tem.2009.10.001. 10.1016/j.tem.2009.10.001 19913438

[6] Zhang H, Liu K (2015). Cellular and molecular regulation of the activation of mammalian primordial follicles: Somatic cells initiate follicle activation in adulthood. Human Reproduction Update.

[7] Webber L, Davies M, Anderson R, Bartlett J, European Society for Human, R., Embryology Guideline Group on, P. O. I. (2016). ESHRE Guideline: management of women with premature ovarian insufficiency. Human Reproduction.

[8] Maclaran K, Panay N (2015). Current concepts in premature ovarian insufficiency. Womens Health (Lond)..

[9] van Kasteren YM, Schoemaker J (1999). Premature ovarian failure: A systematic review on therapeutic interventions to restore ovarian function and achieve pregnancy. Human Reproduction Update.

[10] Dragojevic-Dikic S, Rakic S, Nikolic B, Popovac S (2009). Hormone replacement therapy and successful pregnancy in a patient with premature ovarian failure. Gynecological Endocrinology.

[11] Kawamura K, Cheng Y, Suzuki N, Deguchi M, Sato Y, Takae S (2013). Hippo signaling disruption and Akt stimulation of ovarian follicles for infertility treatment. Proceedings of the National Academy of Sciences of the United States of America.

[12] Vo KCT, Kawamura K (2021). In Vitro Activation Early Follicles: From the Basic Science to the Clinical Perspectives. International Journal of Molecular Sciences.

[13] Lee HN, Chang EM (2019). Primordial follicle activation as new treatment for primary ovarian insufficiency. Clinical and Experimental Reproductive Medicine.

[14] Salem HK, Thiemermann C (2010). Mesenchymal stromal cells: Current understanding and clinical status. Stem Cells.

[15] Zhao YX, Chen SR, Su PP, Huang FH, Shi YC, Shi QY, Lin S (2019). Using mesenchymal stem cells to treat female infertility: An update on female reproductive diseases. Stem Cells International.

[16] Jiao W, Mi X, Qin Y, Zhao S (2020). Stem cell transplantation improves ovarian function through paracrine mechanisms. Current Gene Therapy.

[17] Araujo AB, Salton GD, Furlan JM, Schneider N, Angeli MH, Laureano AM (2017). Comparison of human mesenchymal stromal cells from four neonatal tissues: Amniotic membrane, chorionic membrane, placental decidua and umbilical cord. Cytotherapy.

[18] Mei Q, Mou H, Liu X, Xiang W (2021). Therapeutic potential of humscs in female reproductive aging. Frontiers in Cell and Developmental Biology.

[19] Xie Q, Liu R, Jiang J, Peng J, Yang C, Zhang W (2020). What is the impact of human umbilical cord mesenchymal stem cell transplantation on clinical treatment?. Stem Cell Research & Therapy.

[20] Ding L, Yan G, Wang B, Xu L, Gu Y, Ru T (2018). Transplantation of UC-MSCs on collagen scaffold activates follicles in dormant ovaries of POF patients with long history of infertility. Science China Life Sciences.

[21] Yan H, Zhang J, Wen J, Wang Y, Niu W, Teng Z (2018). CDC42 controls the activation of primordial follicles by regulating PI3K signaling in mouse oocytes. BMC Biology.

[22] Nishi Y, Yanase T, Mu Y, Oba K, Ichino I, Saito M (2001). Establishment and characterization of a steroidogenic human granulosa-like tumor cell line, KGN, that expresses functional follicle-stimulating hormone receptor. Endocrinology.

[23] Zheng W, Zhang H, Gorre N, Risal S, Shen Y, Liu K (2014). Two classes of ovarian primordial follicles exhibit distinct developmental dynamics and physiological functions. Human Molecular Genetics.

[24] Abdi S, Salehnia M, Hosseinkhani S (2013). Steroid production and follicular development of neonatal mouse ovary during in vitro culture. International Journal of Fertility & Sterility.

[25] Devos M, Grosbois J, Demeestere I (2020). Interaction between PI3K/AKT and Hippo pathways during in vitro follicular activation and response to fragmentation and chemotherapy exposure using a mouse immature ovary model. Biology of Reproduction.

[26] John GB, Gallardo TD, Shirley LJ, Castrillon DH (2008). Foxo3 is a PI3K-dependent molecular switch controlling the initiation of oocyte growth. Developmental Biology.

[27] Konala VB, Mamidi MK, Bhonde R, Das AK, Pochampally R, Pal R (2016). The current landscape of the mesenchymal stromal cell secretome: A new paradigm for cell-free regeneration. Cytotherapy.

[28] Kupcova Skalnikova H (2013). Proteomic techniques for characterisation of mesenchymal stem cell secretome. Biochimie.

[29] Caseiro AR, Santos Pedrosa S, Ivanova G, Vieira Branquinho M, Almeida A, Faria F (2019). Mesenchymal Stem/Stromal Cells metabolomic and bioactive factors profiles: A comparative analysis on the umbilical cord and dental pulp derived Stem/Stromal Cells secretome. PLoS One..

[30] Jiao, W., Mi, X., Yang, Y., Liu, R., Liu, Q., Yan, T., et al. (2021). Mesenchymal stem cells combined with autocrosslinked hyaluronic acid improve mouse ovarian function by activating the PI3K-AKT pathway in a paracrine manner. *Stem Cell Research & Therapy*. Accepted. 10.1186/s13287-022-02724-3 PMC8812195 35109928

[31] Jia, Y., Cao, N., Zhai, J., Zeng, Q., Zheng, P., Su, R., et al. (2020). HGF mediates clinical-grade human umbilical cord-derived mesenchymal stem cells improved functional recovery in a senescence-accelerated mouse model of Alzheimer’s disease. *Advanced Science (Weinh).,**7*(17), 1903809. 10.1002/advs.201903809. 10.1002/advs.201903809 PMC7507104 32995116

[32] Nakamura T, Mizuno S (2010). The discovery of hepatocyte growth factor (HGF) and its significance for cell biology, life sciences and clinical medicine. Proceedings of the Japan Academy. Series B, Physical and Biological Sciences.

[33] Ito M, Harada T, Tanikawa M, Fujii A, Shiota G, Terakawa N (2001). Hepatocyte growth factor and stem cell factor involvement in paracrine interplays of theca and granulosa cells in the human ovary. Fertility and Sterility.

[34] Guglielmo MC, Ricci G, Catizone A, Barberi M, Galdieri M, Stefanini M, Canipari R (2011). The effect of hepatocyte growth factor on the initial stages of mouse follicle development. Journal of Cellular Physiology.

[35] Bayer IS (2020). Hyaluronic acid and controlled release: A review. Molecules.

[36] Zheng W, Nagaraju G, Liu Z, Liu K (2012). Functional roles of the phosphatidylinositol 3-kinases (PI3Ks) signaling in the mammalian ovary. Molecular and Cellular Endocrinology.

[37] Grosbois, J., Devos, M., & Demeestere, I. (2020). Implications of nonphysiological ovarian primordial follicle activation for fertility preservation. *Endocrine Reviews, 41*(6). 10.1210/endrev/bnaa020. 10.1210/endrev/bnaa020 32761180

[38] Castrillon DH, Miao L, Kollipara R, Horner JW, DePinho RA (2003). Suppression of ovarian follicle activation in mice by the transcription factor Foxo3a. Science.

[39] Levy O, Kuai R, Siren EM, Bhere D, Milton Y, Nissar N (2020). Shattering barriers toward clinically meaningful MSC therapies. Science Advances.

[40] Zaher W, Harkness L, Jafari A, Kassem M (2014). An update of human mesenchymal stem cell biology and their clinical uses. Archives of Toxicology.

[41] Kumar P, Kandoi S, Misra R, Vijayalakshmi S, Rajagopal K, Verma R (2019). The mesenchymal stem cell secretome: A new paradigm towards cell-free therapeutic mode in regenerative medicine. Cytokine & Growth Factor Reviews.

[42] Zachow R, Uzumcu M (2007). The hepatocyte growth factor system as a regulator of female and male gonadal function. Journal of Endocrinology.

[43] Osuga Y, Tsutsumi O, Momoeda M, Okagaki R, Matsumi H, Hiroi H (1999). Evidence for the presence of hepatocyte growth factor expression in human ovarian follicles. Molecular Human Reproduction.

[44] Gherardi E (1991). Hepatocyte growth factor-scatter factor: Mitogen, motogen, and met. Cancer Cells.

[45] Uzumcu M, Pan Z, Chu Y, Kuhn PE, Zachow R (2006). Immunolocalization of the hepatocyte growth factor (HGF) system in the rat ovary and the anti-apoptotic effect of HGF in rat ovarian granulosa cells in vitro. Reproduction.

[46] Zhang Y, Yan Z, Qin Q, Nisenblat V, Chang H-M, Yu Y (2018). Transcriptome landscape of human folliculogenesis reveals oocyte and granulosa cell interactions. Molecular Cell.

[47] Parrott JA, Skinner M (1999). Kit-ligand/stem cell factor induces primordial follicle development and initiates folliculogenesis. Endocrinology.

[48] Kim JY (2012). Control of ovarian primordial follicle activation. Clinical and Experimental Reproductive Medicine.

[49] Yang W, Zhang J, Xu B, He Y, Liu W, Li J (2020). HucMSC-derived exosomes mitigate the age-related retardation of fertility in female mice. Molecular Therapy.

[50] Gunawardena TNA, Rahman MT, Abdullah BJJ, Abu Kasim NH (2019). Conditioned media derived from mesenchymal stem cell cultures: The next generation for regenerative medicine. Journal of Tissue Engineering and Regenerative Medicine.

[51] Zhang J, Yan L, Wang Y, Zhang S, Xu X, Dai Y (2020). In vivo and in vitro activation of dormant primordial follicles by EGF treatment in mouse and human. Clinical and Translational Medicine.

