# Supplementary material for: HGF Secreted by Mesenchymal Stromal Cells Promotes Primordial Follicle Activation by Increasing the Activity of the PI3K-AKT Signaling Pathway
Source: Stem Cell Rev Rep. 2022 Jan 28;18(5):1834–50. doi: 10.1007/s12015-022-10335-x (PMC9209380; doi:10.1007/s12015-022-10335-x)
Supplement: Supplementary file 1 — Supplementary file1 (DOCX 1124 KB) [file 12015_2022_10335_MOESM1_ESM.docx]

**Supplementary Figures**

**
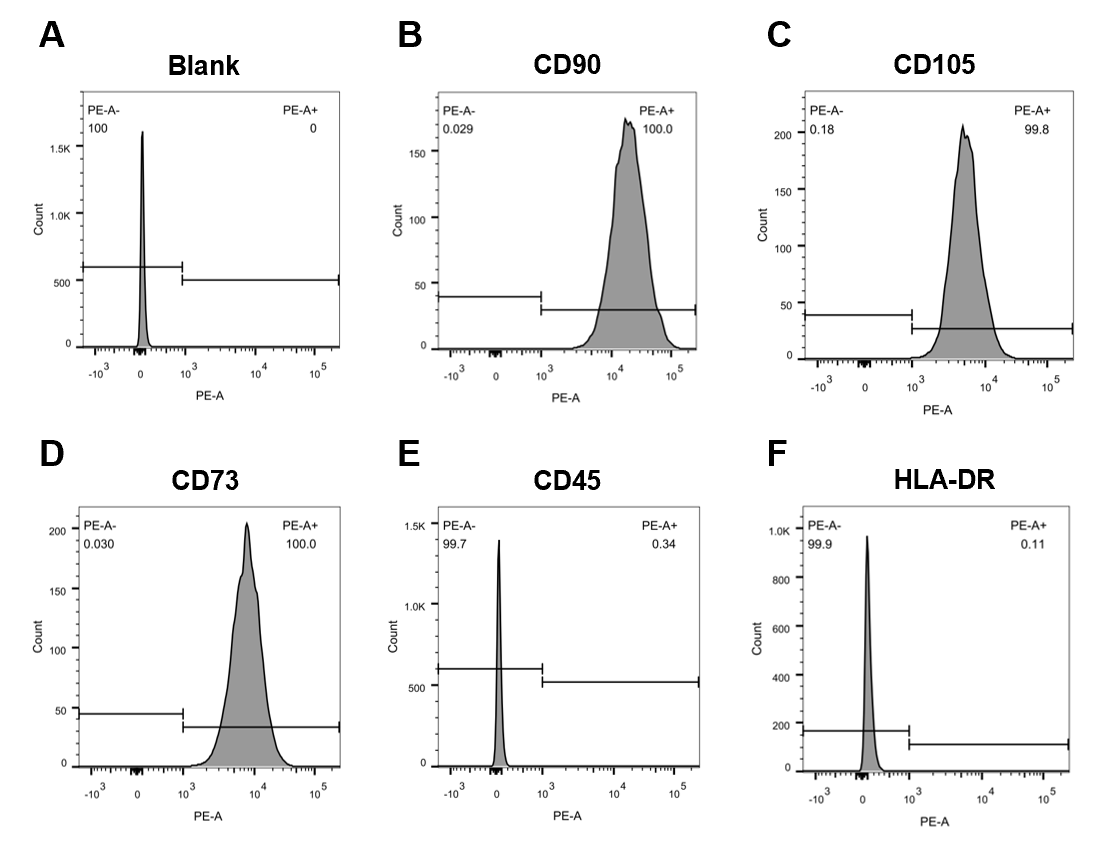
**

Figure S1. **Characterization of hUC-MSCs by flow cytometry analysis.** The hUC-MSCs were positive for CD90, CD105, and CD73, and negative for CD45 and HLA-DR.

**
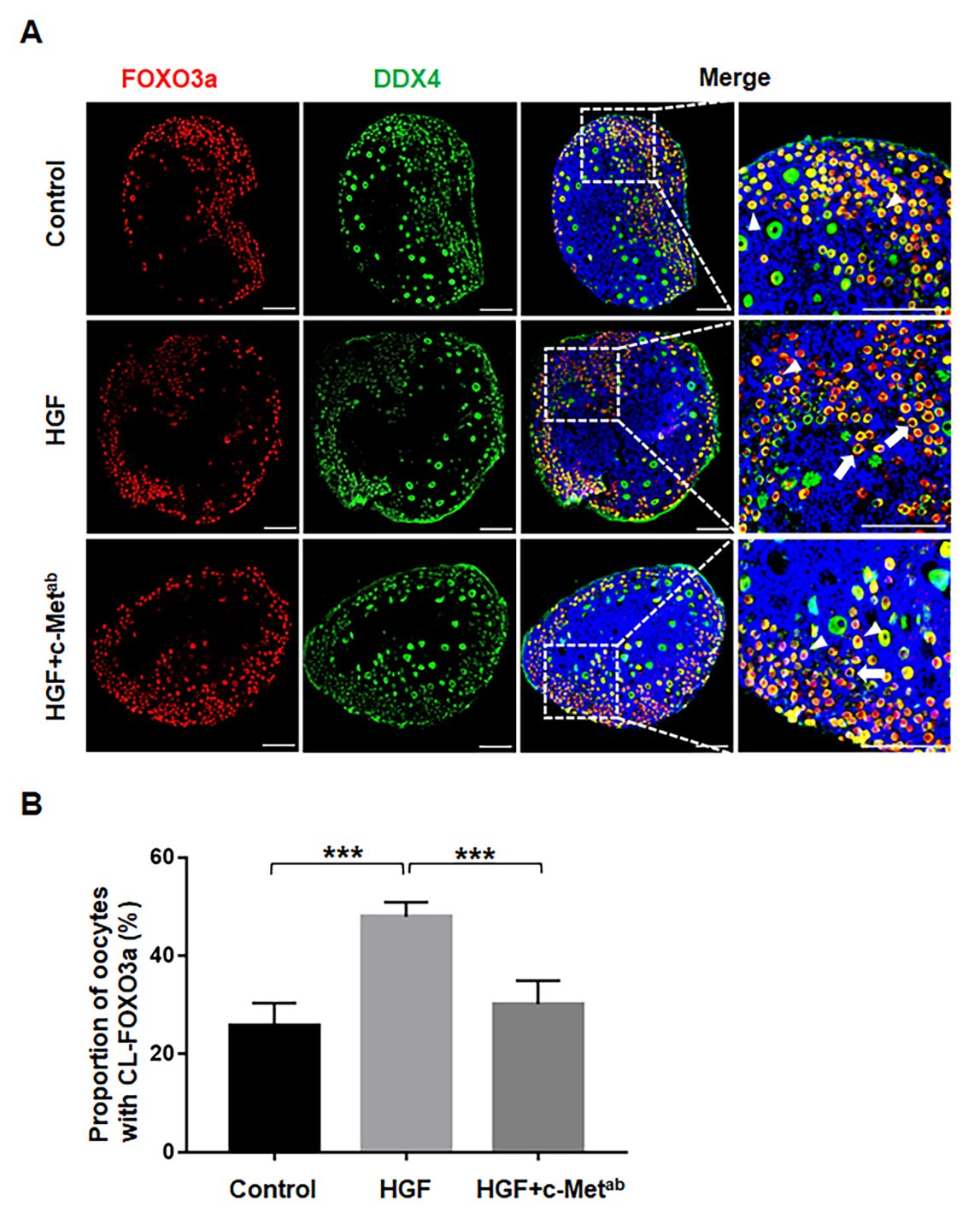
**

Figure S2. **HGF increased the proportion of oocytes with CL-FOXO3a via the HGF receptor c-Met. A.** Immunofluorescence analysis showing the location of FOXO3a in mouse ovaries after 4 days of *in vitro* culture. The ability of HGF to promote the translocation of FOXO3a from the nucleus to the cytoplasm was inhibited by the addition of c-Met^ab^. The arrowheads indicate nuclear localization of FOXO3a, and the arrows indicate the cytoplasmic localization of FOXO3a. **B.** The proportion of CL-FOXO3a was significantly decreased in HGF plus c-Met^ab^-treated ovaries (30.1±4.9%), which was similar to controls (25.8±4.5%), compared to the HGF group (47.9±3.0%). Data are shown as the mean ± SD, n = 5. ****P* < 0.001. Scale bars, 100 μm.
